# LncRNA USP30-AS1 promotes the survival of acute myeloid leukemia cells by *cis*-regulating USP30 and ANKRD13A

**DOI:** 10.1007/s13577-021-00636-7

**Published:** 2021-10-25

**Authors:** Wei Zhou, Shilin Xu, Tingfen Deng, Ruiqing Zhou, Caixia Wang

**Affiliations:** Department of Hematology, School of Medicine, Guangzhou First People’s Hospital, South China University of Technology, Guangzhou, Guangdong China

**Keywords:** USP30-AS1, Acute myeloid leukemia, ANKRD13A, USP30, Methylation

## Abstract

Acute myeloid leukemia (AML) is a malignant tumor derived from leukemia stem cells, with complicated pathogenesis. LncRNAs play an important role in tumors genesis and progression. According to results from bioinformatics analysis, lncRNA USP30-AS1 is highly expressed in AML and both the high expression of USP30-AS1 and low methylation level at Cg03124318 locus of *USP30-AS1* gene promoter are associated with poor prognosis of AML. This study knocked down and overexpressed USP30-AS1 to determine the roles in AML cell lines. High-throughput sequencing was performed to explore the genes regulated by USP30-AS1. Results showed that USP30-AS1 promoted AML cell viability and inhibited apoptosis. Genes regulated by USP30-AS1 are mainly related to genetic regulation and immune system. Among them, *USP30* and *ANKRD13A* genes are close to *USP30-AS1* gene in chromosome. Knockdown of USP30, but not ANKRD13A, abolished the cancer-promoting effects of USP30-AS1. ANKRD13A recognizes Lys-63-linked polyubiquitin chain in HLA-I. USP30-AS1 induced HLA-I internalization from the cell membrane by up-regulating ANKRD13A, which might induce the immune escape of AML cells. ChIP analysis revealed that the regulatory effects of USP30-AS1 on USP30 and ANKRD13A are associated with H3K4me3 and H3K27Ac. In summary, USP30-AS1 probably promotes AML cell survival by *cis*-regulating USP30 and ANKRD13A.

## Introduction

Acute myeloid leukemia (AML) is a malignancy of bone-marrow hematopoietic stem/progenitor cells, including all non-lymphoid acute leukemias, and is the most common type of adult acute leukemia [1]. It has rapid onset, rapid development and serious harm. About 60~70% of patients (especially those under 60 years) die of infection, bleeding and organ infiltration within 1 year of diagnosis and treatment [1, 2]. Therefore, understanding the molecular mechanism of the tumorigenesis and progression of AML can effectively promote clinical diagnosis and the development of new therapies.

Tumor development is a multistage process involving a variety of genetic and extragenetic changes. The changes in the genome can lead to the disruption of the expression of oncogenes and tumor suppressor genes, thus promoting tumor formation [3]. Epigenetics is one of the key areas in the study of genomic function and regulation of gene expression. The main regulatory modes of epigenetic changes include DNA methylation, histone modification, and chromatin remodeling [4]. DNA methylation refers to a process in which organisms use S-adenosine methionine (SAM) as a methyl donor to transfer methyl groups to specific bases under the catalysis of DNA methylation transferase [5]. It has proved that the pathological mechanism of AML closely related to DNA methylation changes and accompanying transcription abnormalities [6]. Toyota et al. studied and analyzed the methylation status of 14 promoter-related CpG islands in 36 cases of AML. Hypermethylation at P15, MDR1, and SDC4 gene promoter was associated with decreased expression of the genes [7]. However, in a study of determining the key epigenetic genes in AML, Hu et al. found that the hypomethylated ZNRF2 gene promotes the expression level of the corresponding mRNA and can be used as a target for AML treatment [8]. Moreover, in a 48-month follow-up study on the treatment of AML patients, it was found that CEBPA methylation status is closely associated with the disease-free survival and overall survival of AML patients. The hypomethylation of CEBPA in leukemia cells can promote the expression of HLA-DR and CD56 and lead to poor prognosis [9]. These studies indicate that abnormal methylation status in gene promoter can be used as an important prognostic biomarker in AML.

As a type of non-coding RNA, LncRNA can regulate gene expression at multiple levels, including transcriptional and post-transcriptional regulation, translation, and post-translational modification, and thus affect a variety of biological functions, such as cell proliferation, differentiation, and apoptosis in cancer [10]. Among them, cis-acting is an important regulatory function of lncRNA, and its knockdown or overexpression will lead to changes in the expression of adjacent genes on the chromosome [11]. At present, relevant studies have shown that some lncRNAs are overexpressed, under-expressed, or even not expressed in AML. Their abnormal expression will cis-regulate the expression of neighboring genes, and then play a role in promoting or suppressing cancer [11, 12]. For example, Fernando et al. found that a novel lncRNA CASC15 was up-regulated in acute lymphoblastic leukemia (B-ALL), and subsequently affected cell survival and proliferation by driving the expression of its adjacent gene SOX4 on the chromosome through the *cis* mechanism, thereby delaying the progression of acute leukemia [12]. In the biological process of lncRNA regulating gene transcription and expression through epigenetic modification, the cis-regulation mechanism plays an important role [11, 13]. The *cis*-regulation is that the regulatory sequences on the same chromosome directly regulate the expression of other adjacent genes. It is available at the DNA, RNA, and protein levels. [14, 15]. lncRNAs can regulate the expression of genes within 1 million bps nearby by cis-acting [14, 16]. In the process of transcription regulation, lncRNA can not only bind DNA sequence, transcription factor, and RNA polymerase complex to affect transcription regulation, but also regulate the transcription process through histone modification [14, 16]. For example, some lncRNAs can regulate gene transcription level *cis* or *trans* by interacting with polymerase II complex [16]. Wang et al. found that an lncRNA LAMP5-AS1 can directly bind DOT1L to promote the activity of methyltransferase and the overall methylation level of intracellular H3K79, thereby regulating the self-renewal program and differentiation block of MLL leukemia cells [17]. This also shows that the regulation of epigenetic modification of lncRNA has been involved in the pathological process of many cancers.

USP30-AS1 is the newly discovered lncRNA transcribed from the antisense strand of the USP30 gene. At present, the regulatory role of USP30-AS1 in cancer has not yet in-depth studied, but its possible involvement in autophagy has mentioned in bladder cancer, which may act as a potential prognostic indicator [18]. USP30, a member of the ubiquitin-specific protease family, is a novel mitochondrial deubiquitinase involved in p53 stability and regulation of a variety of cellular processes [19, 20]. USP30 can deubiquitinate and stabilize mitochondrial division protein DRP1, promote mitochondrial morphology, and thus regulate lipid metabolism and the occurrence of liver cancer [21]. However, the biological function of USP30 in AML is still unclear.

ANKRD13A is an ubiquitin-binding protein, specifically recognizing Lys-63-linked polyubiquitin chain in protein. Different from Lys-48-linked polyubiquitin, Lys-63-linked polyubiquitin more likely impacts the functions of protein than protein degradation [22, 23]. For example, ANKRD13A binds to Lys-63-linked polyubiquitin chain in Cav-1 protein, inducing the transportation of Cav-1 to endosomes [23]. In addition, ANKRD13A induces the internalization of ligand-activated EGFR from cell membrane after binding to Lys-63-linked polyubiquitin chain [22]. A large number of data proves that tumor cells can escape host immunity for sustainable survival [24, 25]. HLA-I is a part of MHC-1 class molecules. Tumor cells with the loss of HLA-I antigen in cell surface cannot be recognized and attacked by CTL or NK cells, leading to immune escape of the tumor [26, 27]. Cancer cells have the ability to induce the internalization of HLA-I on the cell surface, which leads to a decrease in antigen presentation, thereby promoting immune escape and disease progression [25, 26]. However, it is unclear whether ANKRD13A induces the internalization of HLA-I from AML cell membrane.

Here, we used bioinformatics technology to characterize the roles of USP30-AS1 in AML. Furthermore, we knocked down and overexpressed USP30-AS1 to determine the functions in AML cell lines. High-throughput sequencing was performed to explore the gene regulated by USP30-AS1. We also explored the interaction of USP30-AS1 with the adjacent genes USP30 and ANKRD13A to reveal the mechanism underlying the carcinogenic role of USP30-AS1 in AML.

## Materials and methods

### Bioinformatics analysis

The GEPIA database integrates current cancer genomics data, including TCGA cancer data (9736 tumor samples) and GTEx normal tissue data (8587 normal samples) [28]. The GEO database, a public repository for various high-throughput experimental data, can detect mRNA, genomic DNA and protein abundance. In this study, the relevant functions of USP30-AS1 and the methylation level in its promoter were analyzed online through GEPIA and GEO websites, so as to clarify the correlation between USP30-AS1 and the prognosis of AML. The main parameters in the difference analysis were composed of log_2_ values (> 1 or < − 1) and *p* values (< 0.05) indicating.

### Cell culture and grouping

The AML cell lines (HL-60, KG-1, OCI-AML3, Kasumi-1 and THP1) obtained from Shanghai Cell Library of Chinese Academy of Sciences (Shanghai, China) and Typical Cultures Preservation Center (ATCC, MD, USA). The defrosted cell suspensions were inoculated in sterile flask and cultured in RPMI 1640 medium (Gibco, USA) containing 10% fetal bovine serum. During the culture process, the cell culture flask placed in a constant temperature incubator at 37 ℃, 5% CO_2,_ and 95% humidity to ensure asepsis during the whole process. The medium was changed every 2–3 days. After sub-cultured three times, cells used for subsequent experiments. Cell lines with the highest and lowest USP30-AS1 expression selected for loss and gain tests, namely, USP30-AS1 knock down (KD) and overexpression (OE).

### Recombinant plasmid construction and transfection

The RNA sequences of USP30-AS1, USP30, ACACB, and ANKRD13A were obtained from Genebank. Given USP30-AS1 might be partially located in cell nucleus, we silenced USP30-AS1 using CRISPR/Cas9 technology. We used the CRISPR online design tool website (http://www.crisprlnc.org and http://crispr.mit.edu) to design paired sgRNAs that knock down the *USP30-AS1* gene. Selected sgRNAs with higher scores and constructed them into CRISPR-Cas9-related plasmid vectors (GenePharma, Shanghai, China). USP30, ACACB, ANKRD13A, and ASH2L were knocked down by siRNAs (USP30-siRNA: 5′–TTTAGGGTTGTGTTGACTAGG–3′, ACACB-siRNA: 5′–TAAAGTCGCCTCGGATGGACA–3′, ANKRD13A-siRNA: 5′–TTTATGTCGGAGTAAGACTCG–3′, ASH2L-siRNA: 5′–CCCATTGGAACACCCGTTTAA–3′), and the siRNAs were also synthesized by GenePharma Company (Shanghai, China).

In addition, primers were designed according to the full-length sequence of the gene to construct overexpression vectors. The PCR products of USP30-AS1, USP30-AS1_250–550_ (a specific region of the lncRNA: 250~550 bp), USP30, ANKRD13A, and ASH2L genes were mixed with PCDNA3.1 vector, respectively. After digestion with restriction endonuclease Hindii/Xhoi (Thermo Fisher Scientific) and ligation with T4 ligase (Thermo Fisher Scientific) overnight, the mixtures were transformed into *E. coli* DH5α competent cells for expansion culture. Finally, after positive colony screening and PCR identification, the construction of the overexpression vector plasmid was completed.

Before plasmid transfection, the well-growing cells were seeded in a 6-well culture plate at a density of 1 × 10^6^ cells/mL and cultured with serum-containing optimi-mem medium. After the cells were cultured to 70% concentration, the recombinant plasmids of USP30-AS1, USP30, ACACB, ANKRD13A, and ASH2L were transfected into AML cells according to the instructions of the Lipofectamine 2000 manufacturer, and cultured in the complete medium. After 24–48 h of transfection, the expression of GFP in each group of transfected cells was detected under a fluorescence microscope. Finally, the cells were collected for subsequent experiments.

### RNA preparation and quantification

Total RNA was extracted from the cells with TRIZOL reagent (Invitrogen Inc., Carlsbad, USA), and its integrity was checked by agarose gel electrophoresis. Then, cDNA was synthesized according to the instructions of PrimeScriptTM Kit (Takara, Dalian, China). ABI7500 qPCR instrument (ABI Company, Oyster Bay, NY, USA) was used for RT-qPCR detection, and the relative expression of lncRNA and mRNA was normalized to the expression of GAPDH. The primer sequences are shown in Table 1.

**Table 1:** Primer information in PCR assay

Gene names	Direction	Sequence(5′–3′)	*T*_m_ (℃)
USP30-AS1	Forward	GTCTCCCCAGGTCTGTGCTTAA	61.5
	Reverse	GTATTTTTTCCTTATGCTGCCAAA	61.2
USP30	Forward	AGAAAGAAGCGTAGAAAAGGGC	60.6
	Reverse	GCTTGCATCTAAGACCTCATCAT	60.2
ACACB	Forward	CAAGCCGATCACCAAGAGTAAA	60.3
	Reverse	CCCTGAGTTATCAGAGGCTGG	61.3
ANKRD13A	Forward	TGCACCTCCTAGTCTGGAAAA	60.4
	Reverse	AGATGCAATAATGTTCGACCTCG	60.7
TMEM119	Forward	CGGCCTATTACCCATCGTCC	61.9
	Reverse	CTGGGCTAACAAGAGAGACCC	61.5
SSH1	Forward	ACCTTCTGCGTTGCGAAGAC	63
	Reverse	AGGTGGATTTTCGTGTCGCTC	62.7
GAPDH	Forward	GGAGCGAGATCCCTCCAAAAT	61.6
	Reverse	GGCTGTTGTCATACTTCTCATGG	60.9

### CCK-8 assay

CCK-8 kit (Dojindo Molecular Technologies, Inc., Shanghai, China) was used to detect cell viability. The transfected cells were seeded in a 96-well plate at a concentration of 1 × 10^4^ cells/ml, and each experiment was repeated 5 wells. After incubating at 37 °C for 0, 24, 48 or 72 h, add 10 µL of CCK-8 solution to each well and incubate for 1 h. The optical density (OD) value was measured with a microplate reader (Promega Corporation, Fitchburg, WI, USA) at a wavelength of 450 nm.

### Flow cytometry

Collect each group of test cells, wash the cells with pre-cooled PBS, add 300 μl of binding buffer to resuspend the cells, and adjust the cell density to 1 × 10^6^ cells/ml. According to the manufacturer’s recommendations, Annexin V-FITC/PI Apoptosis Detection Kit (TransGen Biotech) was used for apoptosis analysis. Take 100ul of cell suspension into the flow tube, and add 5ul each of Annexin V-FITC and PI. After mixing, incubate at room temperature for 15 min in the dark, and add 400 ul of PBS to the reaction tube. Ensure that the cell apoptosis detected by flow cytometry within 1 h. The ModFit software (Verity Software House) used to analyze flow cytometry data.

### RNA sequencing and data analysis

For cellular RNA-seq with USP30-AS1 knockdown, total RNA was isolated and DNase I was treated using the Maxwell Simply RNA Kit (Promega). 1 μg of quality-verified RNA was used for library preparation and sequenced on Illumina Nextseq 500 (75 bp single-ended mode, 1 × 10^6^ reads/sample). 1 μg of quality-verified RNA was used for library preparation and sequenced on Illumina Nextseq 500 (75 bp single-ended mode, 1 × 10^6^ reads/sample). Differential expression analysis performed using edgeR56 in R/Bioconductor, and the following filters were used to select important genes: adjusted *p* value < 0.05 and |log_2_FC|> 0.585.

### Western blot

The collected cells were washed three times with PBS. RIPA buffer (Beyotime Biotechnology) was added to the lysated cells with 15 min incubation at 4 °C. The protein concentration was determined using the BCA Protein Assay Kit (Beyotime Biotechnology). The loading buffer was added to each protein samples to configure the reaction system. After the proteins were loaded, SDS/PAGE electrophoresis was used to separate the proteins into different bands, and the protein bands were transferred to PVDF membrane by wet transfer method. The membrane was sealed in skim milk powder for 2 h, and the primary antibodies, including anti-USP30 (Dilution: 1:200; abcam, Shanghai, China), anti-ACACB (Dilution: 1:500; Abcam), anti-ANKRD13A (Dilution: 1:500; Abcam), Anti-HLA Class 1 (Dilution: 1:500; Abcam) and GAPDH (Dilution: 1:1000; Abcam), were added with incubation overnight at 4℃. The corresponding secondary antibody was added, followed by the addition of ECL (Perkin Elmer) chemiluminescence chromogen solution. Results analyzed by gel imaging equipment. All bands were identified by ImageJ software in western blot analysis.

### Fluorescence in situ hybridization (FISH) analysis

In FISH analysis, the probe for the detection of USP30-AS1 was designed and synthesized by RiboBio (Guangzhou, China). According to the manufacturer's instructions, FISH detection was performed using a FISH kit (RiboBio). At 42 °C, the cells were treated with 250 μL of pre-hybridization solution (RiboBio) for 1 h, and 250 μL of probe hybridization solution (300 ng/mL) before the incubation overnight. A 4′,6-dimidyl-2-phenylindole (DAPI) diluted in PBS/Tween-20 (1:800) was used for nuclear staining. The cells were observed under a fluorescence microscope (Olympus, Tokyo, Japan) in five different fields.

### Immunofluorescence staining

Cells were inoculated on 4-well slides (Merck Millipore, Darmstadt, Germany) at a density of 1 × 10^4^ cells per well and transfected with 1 μg of the corresponding recombinant CRISPR/Cas9-sgRNA, siRNAs or pcDNA3.1 plasmid. After the transfection for 48 h, the cells were collected and fixed in ice-cold 4% paraformaldehyde (PBS configuration, containing 0.05% Triton X-100) for 10 min. Then, cells were washed with 1 × PBS 3 times, mixed with 2% bovine serum albumin (BSA) to block for 1 h, and incubated with primary anti-HLA Class 1 antibody (Dilution: 1:200; Abcam) at 4 °C overnight. Washed with PBS twice, cells were incubated with FITC-labeled secondary antibody at 37 °C for 5 h, followed by counterstaining with DAPI. The cells were then analyzed using a confocal fluorescence microscope.

### Co-immunoprecipitation (Co-IP)

The ProteiA/G immunoprecipitation magnetic bead kit (Beyotime Biotechnology, Shanghai, China) was used for the immunoprecipitation testing, and the specific steps of the test were operated according to the kit instructions. Breifly, anti-HLA Class 1 antibody was added in each cell lysate and incubated overnight at 4 °C with rotation. The normal human IgG protein was used as a negative control. Protein A/G agarose beads (Beyotime Biotechnology) were then added for affinity binding of primary antibody by a 2 h-incubation at 4 °C with gentle rotation. The magnetic bead–antibody–antigen complex was separated and resuspended in 20 µl SDS-PAGE loading buffer (1×) to detect the ANKRD13A level in the complex.

### Chromatin immunoprecipitation (ChIP) assay

The lysed cells were mixed with cold shearing buffer (Roche Applied Science) containing protease inhibitors, and then treated with sonication to obtain a fragment size of 150–1000 bp DNA. Immunoprecipitation was performed using antibodies against H3K4me1, H3K4me3 and H3K27Ac proteins (ProteinTech) and normal rabbit IgG (Cell Signaling Technology). The DNA sequences of the USP30 and ANKRD13 gene promoter regions in the DNA–protein (H3K4me1, H3K4me3, H3K27Ac and IgG) complexes were measured by qPCR (primers shown in Table 1).

### RNA pull-down assay

To determine the ASH2L binding sites in USP30-AS1, the full length of USP30-AS1, the antisense of USP30-AS1, as well as the specific regions of the lncRNA were synthesized and labeled with biotin using the Biotin RNA Labeling Mix (Roche, Basel, Switzerland) and the Riboprobe Systems with T7 RNA polymerase (Promega, Madison, WI, USA). For the RNA pull-down assay, 3 µg of the purified biotin-labeled RNA probes were incubated with chondrocyte lysates for 4 h at room temperature and subsequently with streptavidin magnetic beads (Thermo Fisher Scientific) overnight at 4 °C. The bound proteins in the pull-down product were analyzed by western blot using ASH2L antibody.

### Statistical analysis

All the experiments were repeated at least three times, without special statement. All statistical data were displayed as means SD and were analyzed using SPSS 17.0 (IBM). The significance of the differences between two groups analyzed with Student’s *t* test, while the differences among three or more groups were conducted using one-way ANOVA with Bonferroni *t* post-test (*p < 0.05, **p < 0.01, ***p < 0.001).

## Results

### Up-regulation of USP30-AS1 in AML associated with poor prognosis

**Fig. 1:**
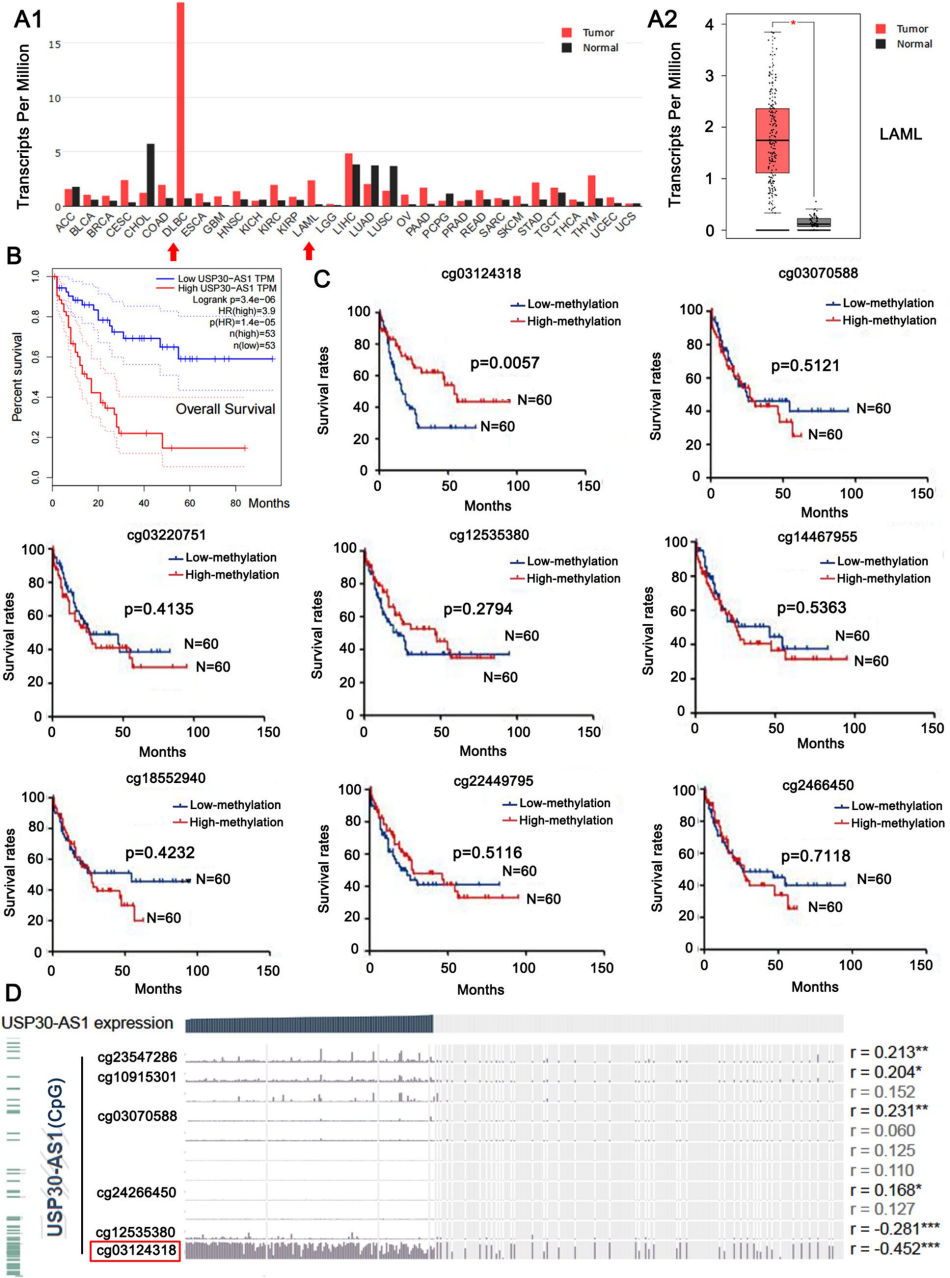
Expression levels of USP30-AS1 and its promoter methylation sites in AML. **A1** Global expression of lncRNA USP30-AS1 across 31 cancer types. **A2** Expression levels of USP30-AS1 between the normal and LAML tumor tissues. *p < 0.05, Tumor tissues: *n* = 173; Normal tissues: *n* = 70. **B** Overall survival analysis between AML patients with low and high expression of USP30-AS1. **C** Effects of low- and high-methylation status across diverse methylation sites of USP30-AS1 promoter on survival in AML patients. *p < 0.05, *n* = 60. **D** Correlation analysis of expression of USP30-AS1 and different methylation status of promoter

Based on the gene expression data in the TCGA and GTEx projects (http://gepia.cancer-pku.cn/), we utilized the bioinformatics technology to study the expression of USP30-AS1 in AML. As shown in Fig. 1A1, among a variety of cancers, USP30-AS1 has the most up-regulated expression in DLBC (Lymphoid Neoplasm Diffuse Large B cell Lymphoma) and LAML (Acute Myeloid Leukemia) tissues. Compared with normal tissues, USP30-AS1 was significantly higher in AML tissues (*p < 0.05, Fig. 1A2). Survival analysis showed that high expression of USP30-AS1 was associated with a lower overall survival rate (***p < 0.001, Fig. 1B), indicating that the high expression of USP30-AS1 was associated with the poor prognosis of AML.

To explore the clinical significance of the epigenetic changes of *USP30-AS1* gene in AML, we detected multiple methylation sites on the *USP30-AS1* gene promoter from the TCGA database (illuminaMethyl450_hg38_GDC). Among them, the low methylation level at Cg03124318 sit (chromosome 12: 109,054,184–109,054,186) is related to the low overall survival rate of AML patients (**p < 0.01, Fig. 1C), but this phenomenon did not appear at other methylation sites. Bioinformatics analysis showed that the methylation level at Cg03124318 was negatively correlated with the expression of USP30-AS1 (*r* = − 0.452, ***p < 0.001, Fig. 1D). It can judge that the hypomethylation of Cg03124318 might lead to the high expression of USP30-AS1 in AML, whereby associating to poor prognosis. Due to ethical reason, we are currently unable to obtain enough bone marrow of AML patients and healthy subjects to identify the epigenetic changes of USP30-AS1, the expression of USP30-AS1 and their association with the prognosis of AML patients. However, results from bioinformatics analysis prompted us to further explore the regulatory effects of USP30-AS1 in AML cell lines.

### USP30-AS1 promotes the viability of AML cells and suppresses apoptosis

**Fig. 2:**
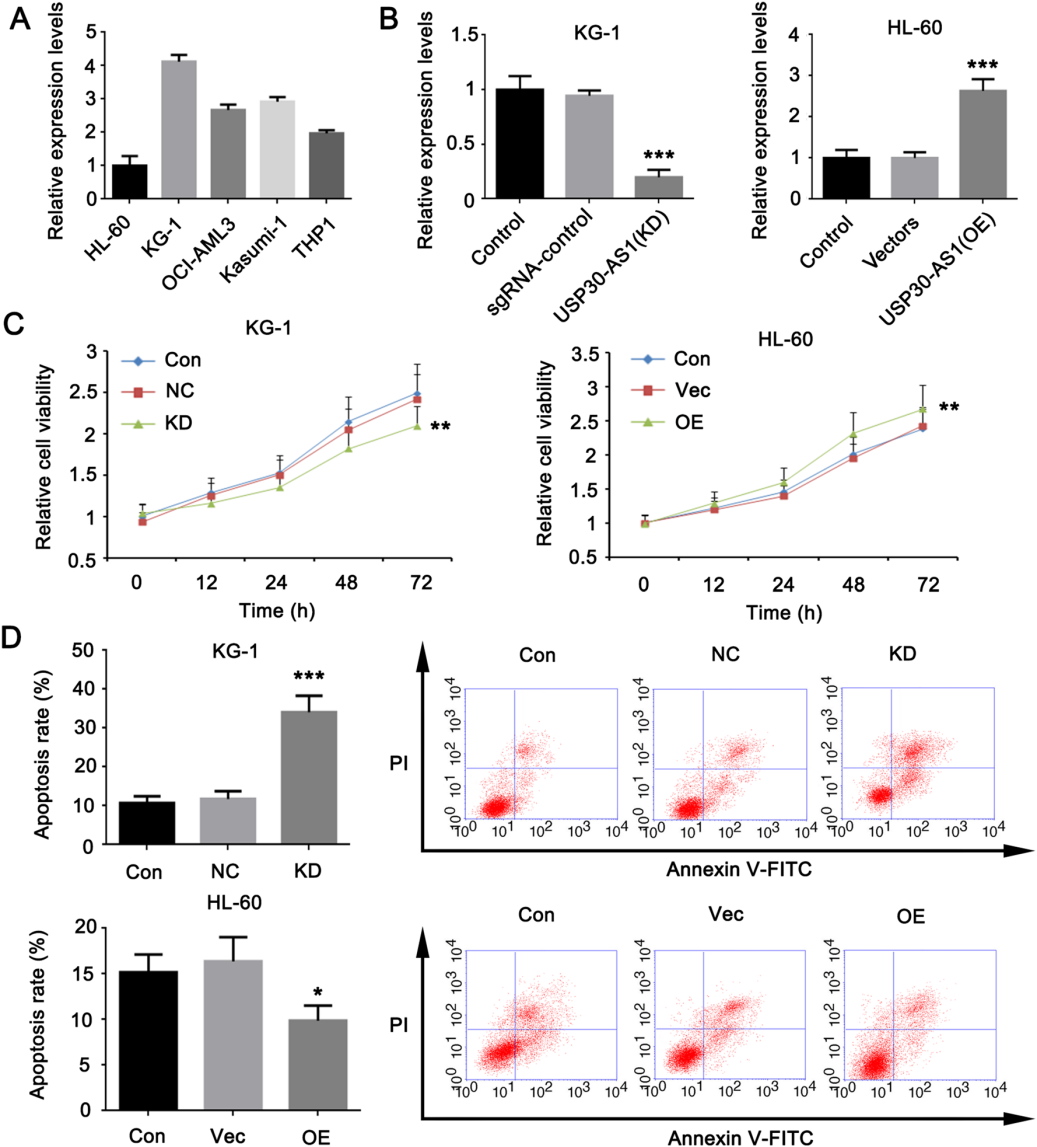
Effect of USP30-AS1 on AML cells after the knockdown and overexpression. **A** Expression levels of USP39-AS1 across five cell lines. Cells with the highest and lowest expression of USP30-AS1 were chose. **B** Expression profiles of USP30-AS1 after the knockdown or overexpression in KG1 and HL-60 cells. **C** AML cell viability was measured using a CCK-8 assay following knockdown and overexpression of USP30-AS1. **D** Apoptosis rates of KG1 and HL-60 cells with knockdown and overexpression of USP30-AS1. Significant differences are indicated by *p < 0.05, **p < 0.01 and ***p < 0.001, *n* = 3

To detect the impact of the high expression of USP30-AS1 on AML cells, we tested 5 cell lines that related to AML (including HL-60, KG-1, OCI-AML3, Kasumi-1, and THP1). As shown in Fig. 2A, among these cell lines, USP30-AS1 has the highest expression in KG-1 cells and the lowest expression in HL-60 cells. For this reason, we selected KG-1 and HL-60 cell lines for loss and gain experimental studies to clarify the effect of USP30-AS1 on the characteristics of AML cells. In the knockdown test of USP30-AS1, we transfected the CRISPR/Cas9-sgRNA vector into KG-1 cells, and found the significant reduction of USP30-AS1 expression (***p < 0.001, Fig. 2B), indicating that USP30-AS1 was successfully knocked down. Transfection with an overexpression vector can promote the expression of USP30-AS1 in HL-60 cells (***p < 0.001, Fig. 2B). The cell viability test and apoptosis results showed that the reduction of USP30-AS1 expression inhibited the proliferation of KG-1 cells and increased the rate of apoptosis. Forcing the expression of USP30-AS1 in HL-60 cells promoted cell viability while reducing the rate of cell apoptosis (*p < 0.05, **p < 0.01, or ***p < 0.001, Fig. 2C and D). It can be seen that the high expression of USP30-AS1 can inhibit AML cell apoptosis and promote the viability of AML cells.

### High-throughput sequencing reveals genes regulated by USP30-AS1

**Fig. 3:**
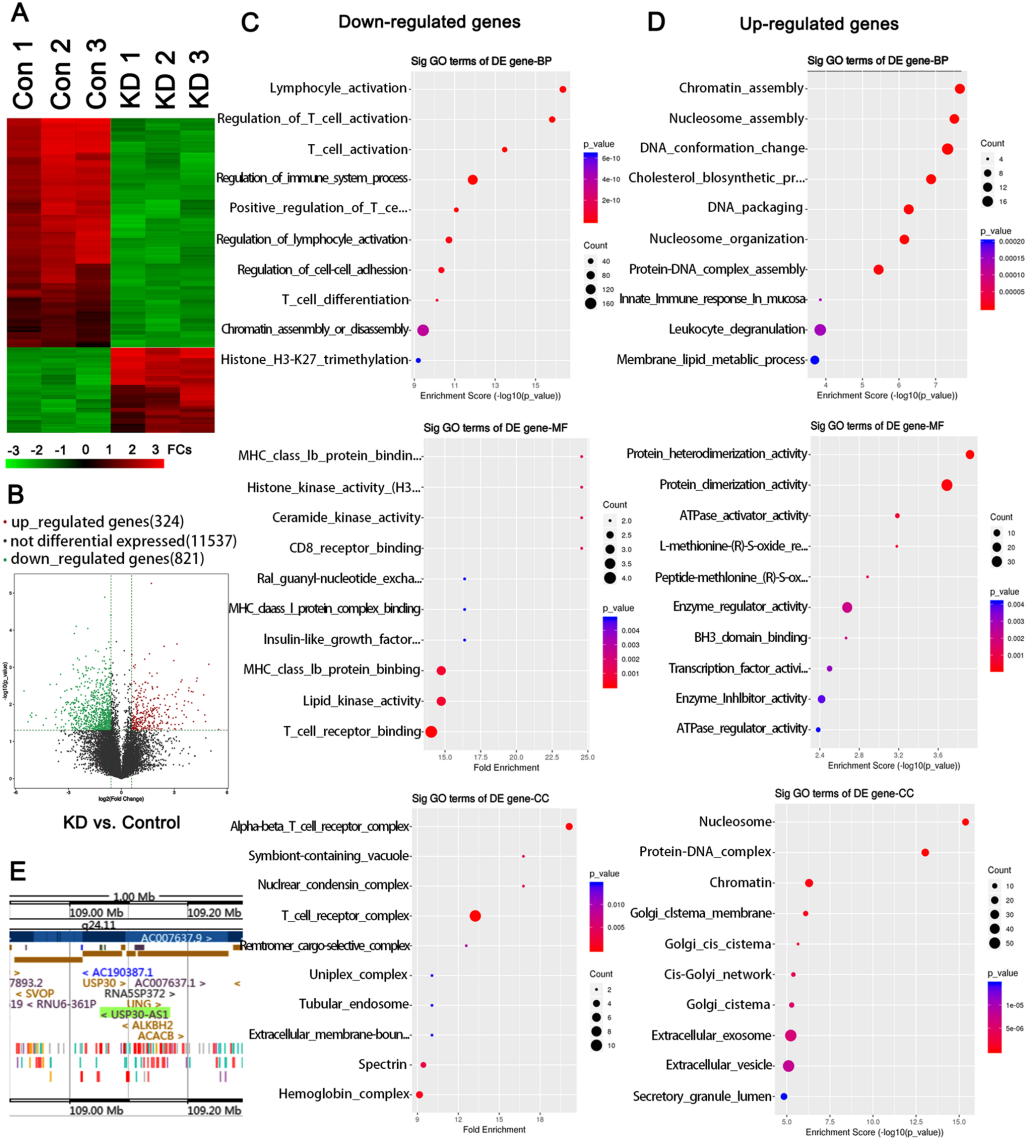
RNA sequencing in KG1cells with USP30-AS1 knockdown or not. **A** Heat map representation of global gene expression changes in the KG1 cells with knockdown of USP30-AS1. **B** Volcano plot of RNA-seq analysis of differentially expressed genes in USP30-AS1 knockdown vs. normal KG1 cells. **C** and **D** USP30-AS1-affected genes in KG1 cells were analyzed and annotated by the Gene Ontology (GO) analysis. **E** Adjacent gens of *USP30-AS1* gene within 1 Mb in the chromosome. Significant differences are indicated by *p < 0.05, *n* = 3

To explore the potential molecular basis and regulatory network of USP30-AS1 in promoting cancer, we performed high-throughput sequencing on KG-1 cells with USP30-AS1 knockdown or not. Results showed that knocking down USP30-AS1 in KG-1 cells resulted in a significant up-regulation of 324 genes (*p < 0.05 and log_2_FC > 0.585) and a significant down-regulation of 821 genes (*p < 0.05 and log_2_FC < -0.585, Fig. 3A and B). GO-functional enrichment analysis revealed that the down-regulated genes mainly related to the immune regulatory system, such as lymphocyte/T cell activation, lipid kinase activity, T cell receptor/ MHC-1b protein binding, etc. (*p < 0.05, **p < 0.01, or ***p < 0.001, Fig. 3C). The enriched functional modules of significantly up-regulated genes were mainly associated with the epigenetic regulation, such as participation in chromatin/nucleosome assembly, DNA conformation changes, protein–DNA complex assembly, protein dimer/heterodimerization's activity regulation, etc. (*p < 0.05, **p < 0.01, or ***p < 0.001, Fig. 3D). *USP30* is the gene that had the most significant down-regulation after USP30-AS1 knockdown. The cancer-promoting effect of USP30 has been confirmed in other types of cancers. Apart from *USP30*, *ANKRD13A* is another one among the top ten down-regulated genes after USP30-AS1 knockdown. ANKRD13A is implicated to the internalization of receptor protein at cell membrane. Since most immune signals are dependent on the binding of ligands to cell receptors, ANKRD13A probably regulates multiple immune responses mentioned in the GO-functional enrichment analysis. Interestingly, both *USP30* and *ANKRD13A* genes are located nearby USP30-AS1 genes. There are other genes which are in the vicinity of the *USP30-AS1* locus, such as the *ACACB*, *TMEM119,* and *SSH1* genes (Fig. 3E). Consistently, their expression was also significantly changed following USP30-AS1 knockdown. Many lncRNAs can regulate the expression of the adjacent genes through *cis* or *trans* mechanism. Combining with the fact that USP30-AS1 is highly expressed in KG-1 cells, we conjectured that USP30-AS1 can regulate these genes in AML, through *cis* or *trans* mechanism.

### USP30-AS1 targets USP30 to promote cancer in AML cells

**Fig. 4:**
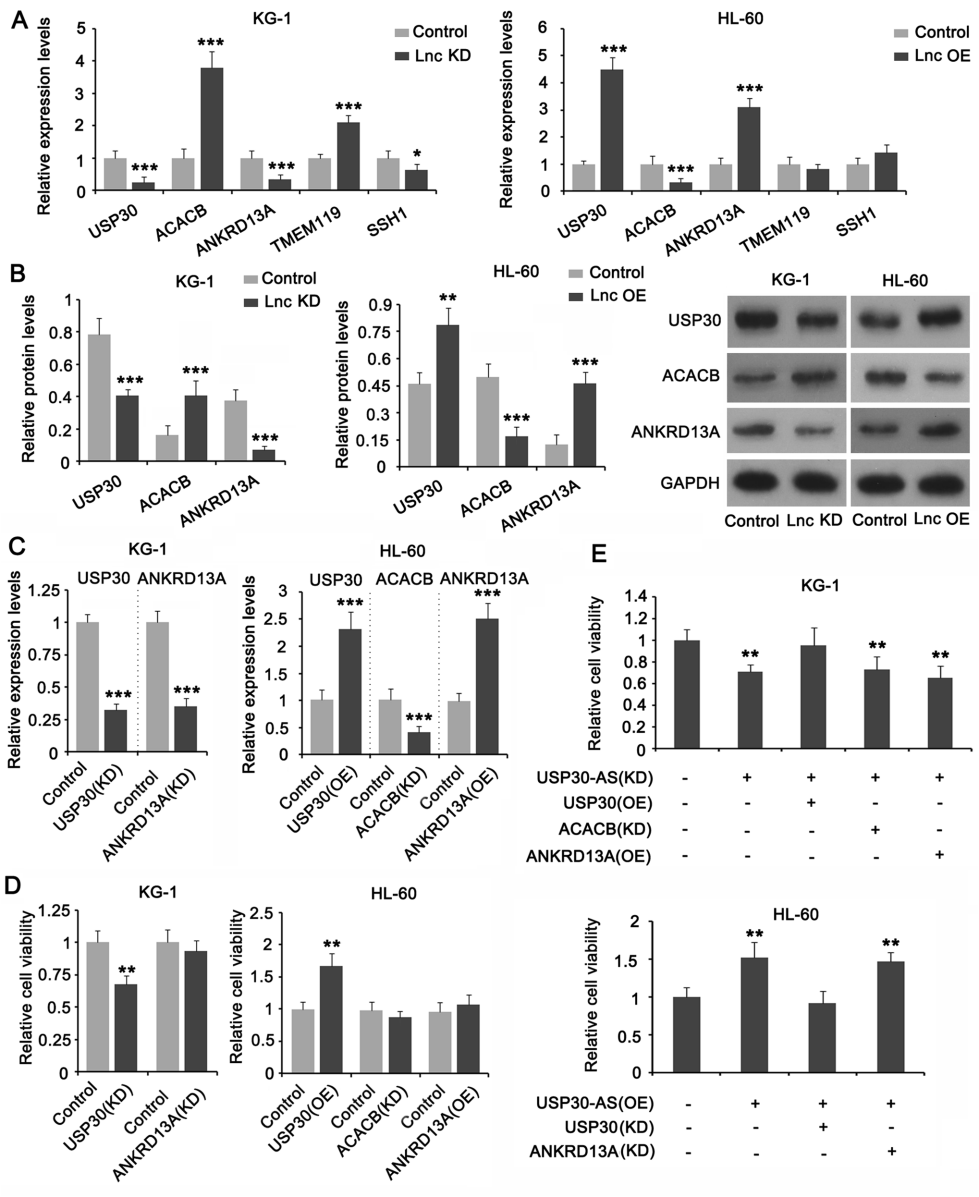
Effects of USP30-AS1 on the expression of the adjacent genes and on AML cells viability. **A** Effects of knockdown and overexpression of USP30-AS1 in KG1 and HL-60 cells on the expression levels of the 5 adjacent genes. **B** Protein expression of USP30, ACACB and ANKRD13A were measured in KG1 and HL-60 cells. **C** Expression levels of USP30, ACACB and ANKRD13A in KG1 and HL-60 cells with knockdown and overexpression treatments. **D** Cells viabilities of USP30, ACACB and ANKRD13A in KG1 and HL-60 cells with knockdown and overexpression treatments. **E** Cells viabilities of USP30-AS1 interacted with USP30, ACACB and ANKRD13A were measured in AML cells transfected with recombinant plasmids, including knockdown and overexpression. Significant differences are indicated by *p < 0.05, **p < 0.01 and ***p < 0.001, *n* = 3

**Fig. 5:**
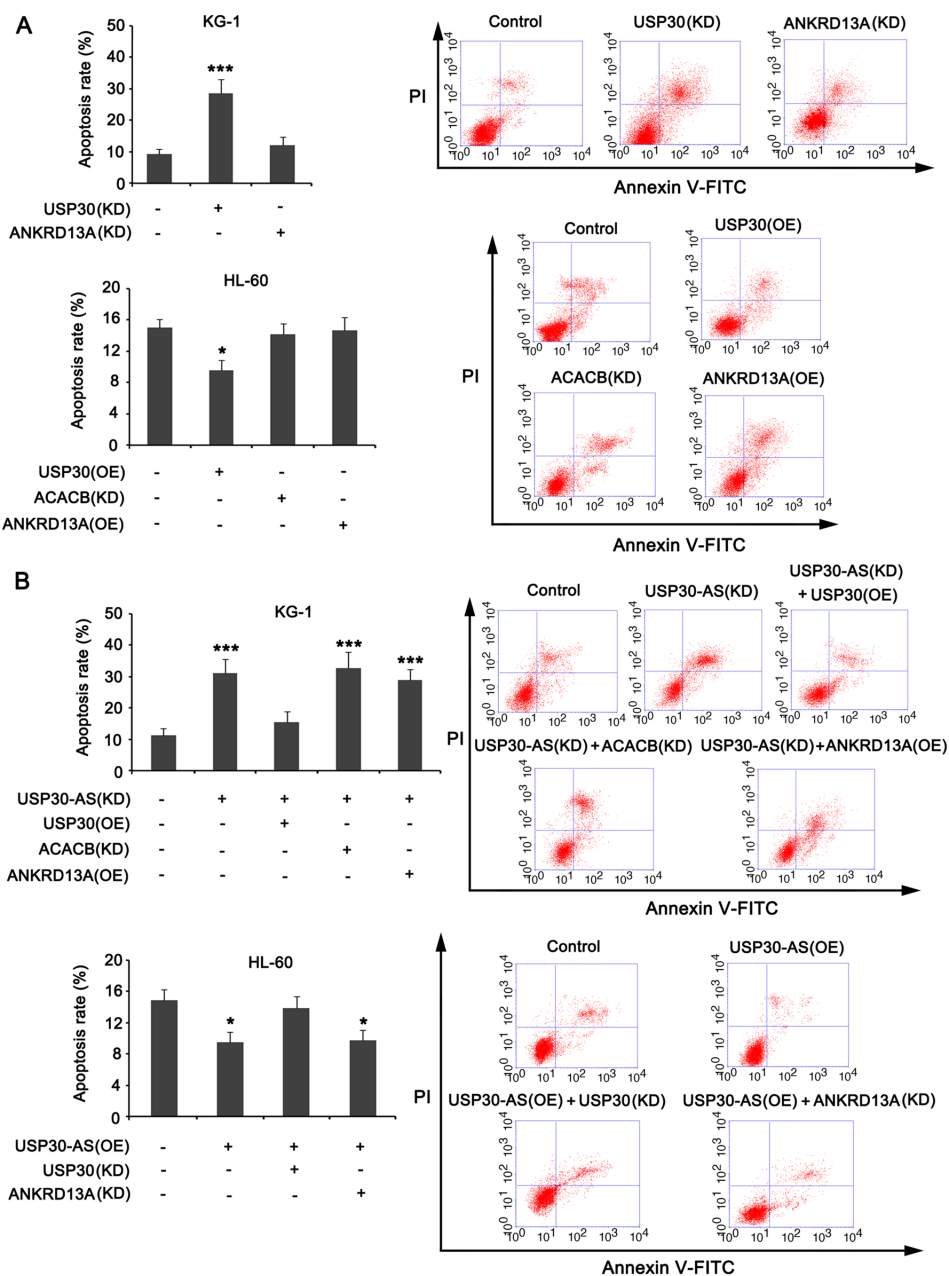
USP30-AS1 cis-regulates the adjacent gene expressions and effects the AML cell apoptosis. **A** Flow cytometry was performed to observe the effects of USP30, ACACB and ANKRD13A knockdown or overexpression on the apoptosis of AML cells. **B** Apoptosis rates of USP30-AS1 interacted with USP30, ACACB and ANKRD13A were measured in AML cells transfected with recombinant plasmids, including knockdown and overexpression. Significant differences are indicated by *p < 0.05, and ***p < 0.001, *n* = 3

We further performed PCR and western blot studies to confirm the regulatory effect of USP30-AS1 on the five adjacent genes (*USP30*, *ACACB*, *ANKRD13A*, *TMEM11*9, and *SSH1*). The expression levels of only three genes (*USP30*, *ACACB,* and *ANKRD13A*) were significantly impacted with the knockdown and overexpression of USP30-AS1 in both KG-1 and HL-60 cells. Among these genes, the expressions of USP30 and ANKRD13A were positively regulated by USP30-AS1, while the expression of ACACB was negatively regulated by USP30-AS1 (*p < 0.05, **p < 0.01, or ***p < 0.001, Fig. 4A and B). We performed a series of loss and gain trials in KG-1 and HL-60 cell lines to detect the regulatory effect of USP30, ACACB, and ANKRD13A in AML cells. PCR results showed that knocking down USP30 and ANKRD13A caused significant reduction of their mRNA levels in KG-1 cells (***p < 0.001, Fig. 4C). Overexpressing the USP30 and ANKRD13A genes in HL-60 cells significantly increased their mRNA levels (**p < 0.01). As the CDS of *ACACB* gene is too long, we are unable to overexpress it. Thus, we only knocked down ACACB, which caused significant reduction in its mRNA expression (Fig. 4C). The results of cell viability test showed that, regardless of gene knockdown or overexpression, only USP30 had a significant inhibitory/promoting effect on the viability of the two AML cells (**p < 0.01, Fig. 4D). Moreover, forcing the expression of USP30 abolished the suppression of KG-1 cell viability caused by UPS30-AS1 knockdown, while overexpression of ANKRD13A and knockdown of ACACB had no such effect (Fig. 4E); Knocking down USP30 abolished the promoting effect of UPS30-AS1 overexpression on HL-60 cell viability. Flow cytometry result showed that only USP30 exerted regulatory effect on apoptosis in AML cells (***p < 0.001, Fig. 5A). In addition, overexpression of USP30 abrogated the regulatory effect of UPS30-AS1 knockdown on apoptosis in KG-1 cells; knockdown of USP30 abrogated the regulatory effect of UPS30-AS1 overexpression on apoptosis in HL-60 cells (Fig. 5B). These results indicate that USP30 mediates the cancer-promoting effect of USP30-AS1 in AML cells.

### USP30-AS1 promotes ANKRD13A to induce the translocation of HLA-1 from the cell membrane to the cytoplasm

**Fig. 6:**
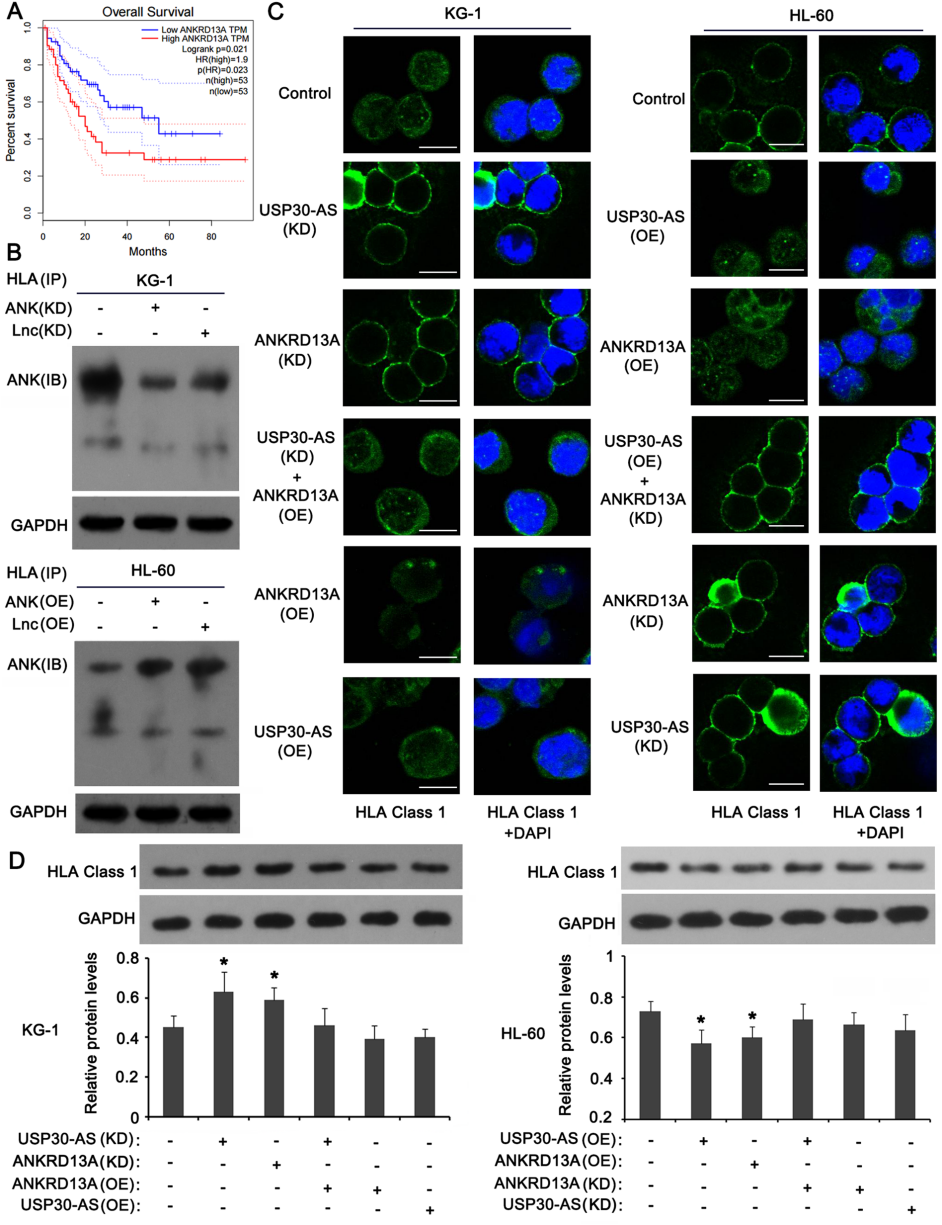
Regulatory role of USP30-AS1 mediates ANKRD13A upregulating to induce HLA-I internalization. **A** Survival analysis between AML patients with low and high expression of ANKRD13A. **B** Co-immunoprecipitation was used to identify interaction between HLA-I and ANKRD13A or SUP30-AS1 in AML cells, including knockdown and overexpression. **C** FISH analysis of the location of HLA-I (green) in the cytomembrane of AML cells across knockdown and overexpression treatments. **D** Protein expressions of HLA-I were measured in AML cells transfected with recombinant plasmids, including knockdown and overexpression. Significant differences are indicated by *p < 0.05, *n* = 3

Although ANKRD13A did not regulate AML cell viability and apoptosis, the survival analysis showed that the high expression of ANKRD13A results in poor prognosis of AML (Fig. 6A). Thus, we speculated that ANKRD13A may affect the development of AML through other pathways. ANKRD13A is an ubiquitin-binding protein that can bind to the ubiquitin linked to'Lys-63' to regulate protein transport. Human Leukocyte Antigen-I (HLA-1) is an important histocompatibility complex (MHC), which plays an important role in the immune response. It has reported that "Lys-63" linked ubiquitin in protein induces the translocation, such as from the cell membrane to the cytoplasm [22, 23]. From this, we conjectured that ANKRD13A regulates the translocation of HLA-1 from the cell membrane to the cytoplasm after binding to "Lys-63" linked ubiquitin in HLA-1. As indicated by immunoprecipitation, knockdown of ANKRD13A or USP30-AS1 decreased the enrichment of ANKRD13A in HLA-1 protein in KG-1 cells; overexpression of ANKRD13A or USP30-AS1 conversely increased the enrichment of ANKRD13A in HLA-1 protein in HL-60 cells (Fig. 6B). As indicated by immunofluorescence test (Fig. 6C), knockdown of ANKRD13A and USP30-AS1 increased the abundance of HLA-1 protein in the cell membrane of KG-1 cells; the effect exerted by USP30-AS1 knockdown was abolished by ANKRD13A overexpression; overexpression of ANKRD13A and USP30-AS1 individually was associated with very low level of HLA-1 protein in the cell membrane of KG-1 cells. Overexpression of ANKRD13A and USP30-AS1 individually decreased HLA-1 protein in the cell membrane in HL-60 cells, compared to control cells; the effect exerted by USP30-AS1 overexpression was abolished by ANKRD13A knockdown; Knockdown of ANKRD13A and USP30-AS1 individually was associated with high level of HLA-1 protein in the cell membrane of HL-60 cells. As indicated by western blot, knockdown of ANKRD13A and USP30-AS1 increased the HLA-1 protein in KG-1 cells (*p < 0.05, Fig. 6D). USP30-AS1 knockdown together with ANKRD13A overexpression, or individual overexpression of ANKRD13A and USP30-AS1 had no effect on HLA-1 protein level in KG-1 cells. Overexpression of ANKRD13A and USP30-AS1 decreased the HLA-1 protein in HL-60 cells (*p < 0.05).

### USP30-AS1 increases the expression of USP30 and ANKRD13A through a *cis* mechanism

**Fig. 7:**
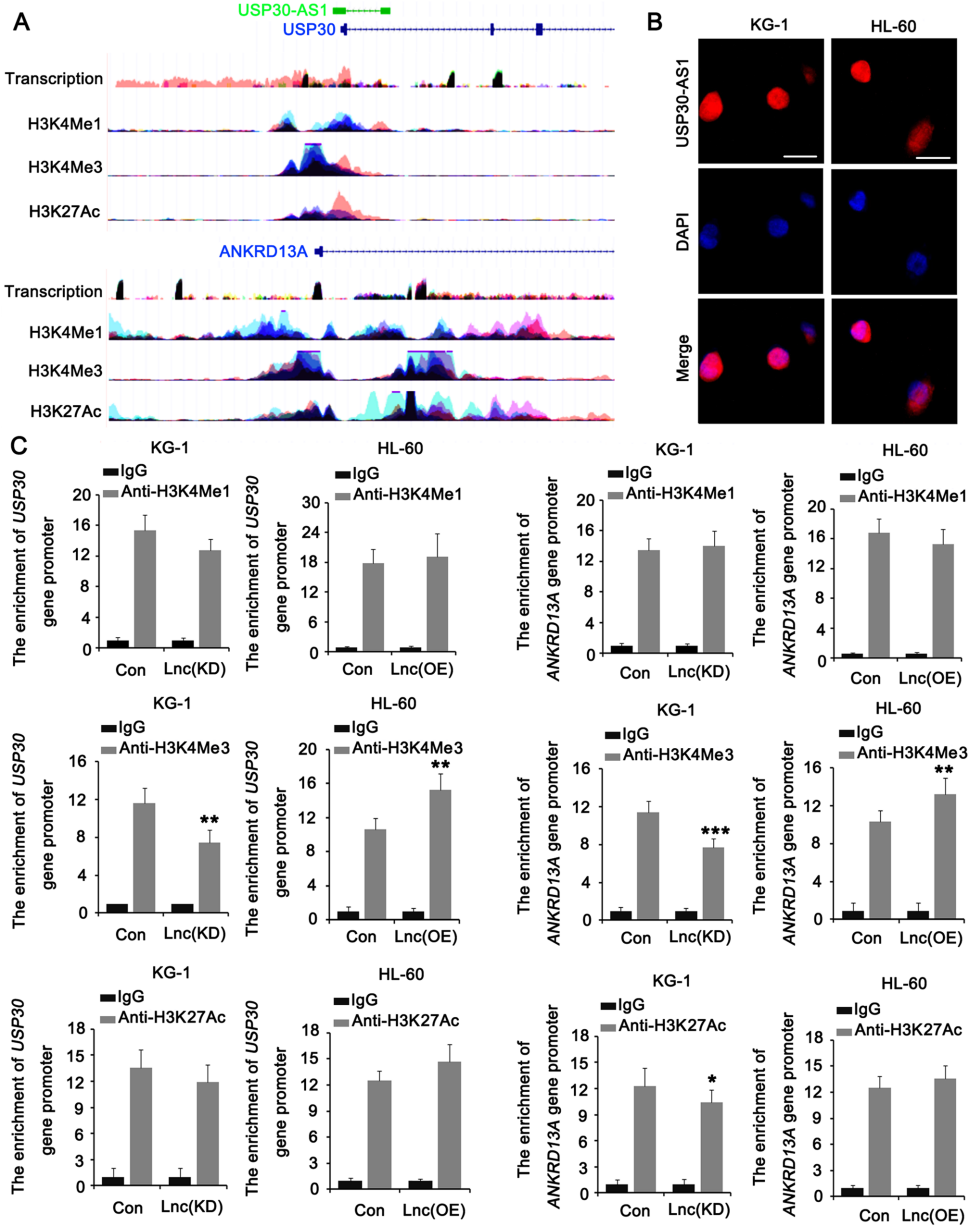
USP30-AS1 regulates USP30 and ANKRD13A in *cis* in AML cells. **A** Schematic illustration of the USP30-AS1 and USP30/ANKRD13A locus, their promoters enriched in the H3K4me1, H3K4me3 and H3K27Ac proteins. **B** FISH analysis of the location of USP30-AS1 (red) in the nuclear of AML cells. **C** ChIP was performed to assess the enrichments of USP30 and ANKRD13A promoters in H3K4me1, H3K4me3 and H3K27Ac proteins. Significant differences are indicated by *p < 0.05, **p < 0.01 and ***p < 0.001, *n* = 3

The previous tests confirmed that USP30-AS1 positively regulates the expression of USP30 and ANKRD13A. We noticed that the locations of the *USP30* and *ANKRD13A* genes in the twelfth chromosome are very close to the location of the *USP30-AS1* gene (Fig. 7A). USP30-AS1 is a lncRNA, which can regulate the expression of its neighboring genes (usually within 1 million bps from the lncRNA gene), that is, cis-acting. With the aim of further exploring the regulatory mechanism of USP30-AS1 on USP30 and ANKRD13A, we designed a FISH test which found that USP30-AS1 was mainly present in the nucleus (Fig. 7B). Bioinformatics analysis (https://www.encodeproject.org/) showed that the *USP30* and *ANKRD13A* gene promoters enriched in all H3K4me1, H3K4me3, and H3K27Ac proteins. This indicated that USP30 and ANKRD13A regulated by histone methylation and acetylation (Fig. 7A). On this basis, we performed ChIP analysis after knockdown or overexpression of USP30-AS1. The results showed that in USP30-AS1 (KD) cells, the enrichment of USP30 and ANKRD13A promoters in H3K4me3 and H3K27Ac proteins significantly reduced. In the USP30-AS1 (OE) group of cells, the enrichment of the USP30 and ANKRD13A promoters in the H3K27Ac protein was significantly increased (*p < 0.05, Fig. 7C).

### ASH2L is implicated in the *cis* regulation of USP30-AS1 in USP30 and ANKRD13A expression

Histone methyltransferase activity is regulated by two counteracting groups of chromatin proteins termed Polycomb group (PcG) and Trithorax group (TrxG) proteins. PcG proteins are specific for the trimethylation of Lys27 on histone H3 (H3K27me3) and repress transcription. By contrast, TrxG proteins are specific for the trimethylation of Lys4 on histone H3 (H3K4me3) and activate transcription. Using a web for RNA–Protein Interaction Prediction (http://pridb.gdcb.iastate.edu/RPISeq/), we analyzed whether there are potential interactions of USP30-AS1 with some PcG and TrxG proteins. Among these PcG and TrxG proteins, ASH2L (a TrxG protein) is the only one that both scores from RF and SVM classifier Predictions are more than 0.85 (Table 2). Therefore, ASH2L is selected for further study. As indicated by catRAPID (http://service.tartaglialab.com/update_submission/392876/94a348ba99), ASH2L is predicted to bind to many regions of USP30-AS1, but there regions are mostly located within the 250–550 bp of USP30-AS1 (Table 3 and Fig. 8A and B). Based on these information, we synthesized the full length of USP30-AS1, the antisense of USP30-AS1, as well as the specific regions of the lncRNA to pull down ASH2L. ASH2L was pulled down by the full length of USP30-AS1, but hardly by the antisense of USP30-AS1 (Fig. 8C). In addition, ASH2L was pulled down by the fragment of USP30-AS1 from 250 to 500 bp, and by other fragments containing the region (250–500 bp). However, fragments without the region (250–500 bp) showed very weak capacity to bind ASH2L. Therefore, the region (250–500 bp) is very important for USP30-AS1 binding to ASH2L. To determine whether ASH2L is implicated in the regulatory effect of USP30-AS1 on USP30 and ANKRD13A expression, we knocked down or overexpressed ASH2L in KG-1 and HL-60 cells (Fig. 8D). As indicated by PCR, the knockdown of ASH2L decreased USP30 and ANKRD13A expression (***p < 0.001) and impaired the promoting effect of overexpressed USP30-AS1 on USP30 and ANKRD13A expression (Fig. 8E). The overexpression of ASH2L increased USP30 and ANKRD13A expression (***p < 0.001) and attenuated the reduction USP30 and ANKRD13A after USP30-AS1 knockdown. To further determine that ASH2L is involved in the *cis* regulation of USP30-AS1, we conducted ChIP assay using H3K4me3 as the IP protein. Overexpression of ASH2L partially restored the enrichment of USP30 and ANKRD13A promoters in H3K4me3 protein after USP30-AS1 knockdown (Fig. 8F). Similar to USP30-AS1, overexpression of a fragment of USP30-AS1 (250–550 bp) also increased the enrichment of USP30 and ANKRD13A promoters in H3K4me3 protein (*p < 0.05). ASH2L knockdown dramatically decreased the enrichment of USP30 and ANKRD13A promoters in despite USP30-AS1 overexpression.

**Table 2:** Prediction of the binding of USP30-AS1 to PcG and TrxG protein complexes

Protein complex	Prediction using RF classifier	Prediction using SVM classifier
PcG		
EZH2	0.75	0.86
EED	0.85	0.71
PCGF2	0.75	0.9
TrxG		
MLL1/KMT2A	0.75	0.89
WDR5	0.7	0.93
ASH2L	0.9	0.88

**Table 3:** Prediction of the binding of USP30-AS1 to ASH2L

#	Protein region	RNA region	Interaction propensity	Discriminative power	Normalized score
1	450–501	284–335	12.91	35	3.12
2	375–426	284–335	12.26	35	2.97
3	25–76	284–335	11.83	33	2.86
4	451–502	284–335	10.9	32	2.64
5	376–427	284–335	10.9	32	2.64
6	450–501	301–352	10.89	32	2.64
7	151–202	284–335	10.57	32	2.56
8	450–501	451–502	10.34	32	2.51
9	450–501	276–327	9.98	28	2.42
10	251–302	284–335	9.95	28	2.42
11	126–177	284–335	9.95	28	2.42
12	450–501	309–360	9.85	28	2.39
13	125–176	284–335	9.84	28	2.39
14	450–501	401–452	9.81	28	2.38
15	25–76	301–352	9.8	28	2.38
16	375–426	301–352	9.79	28	2.38
17	375–426	451–502	9.76	28	2.37
18	375–426	276–327	9.63	28	2.34
19	25–76	451–502	9.39	28	2.28
20	176–227	284–335	9.38	28	2.28

**Fig. 8:**
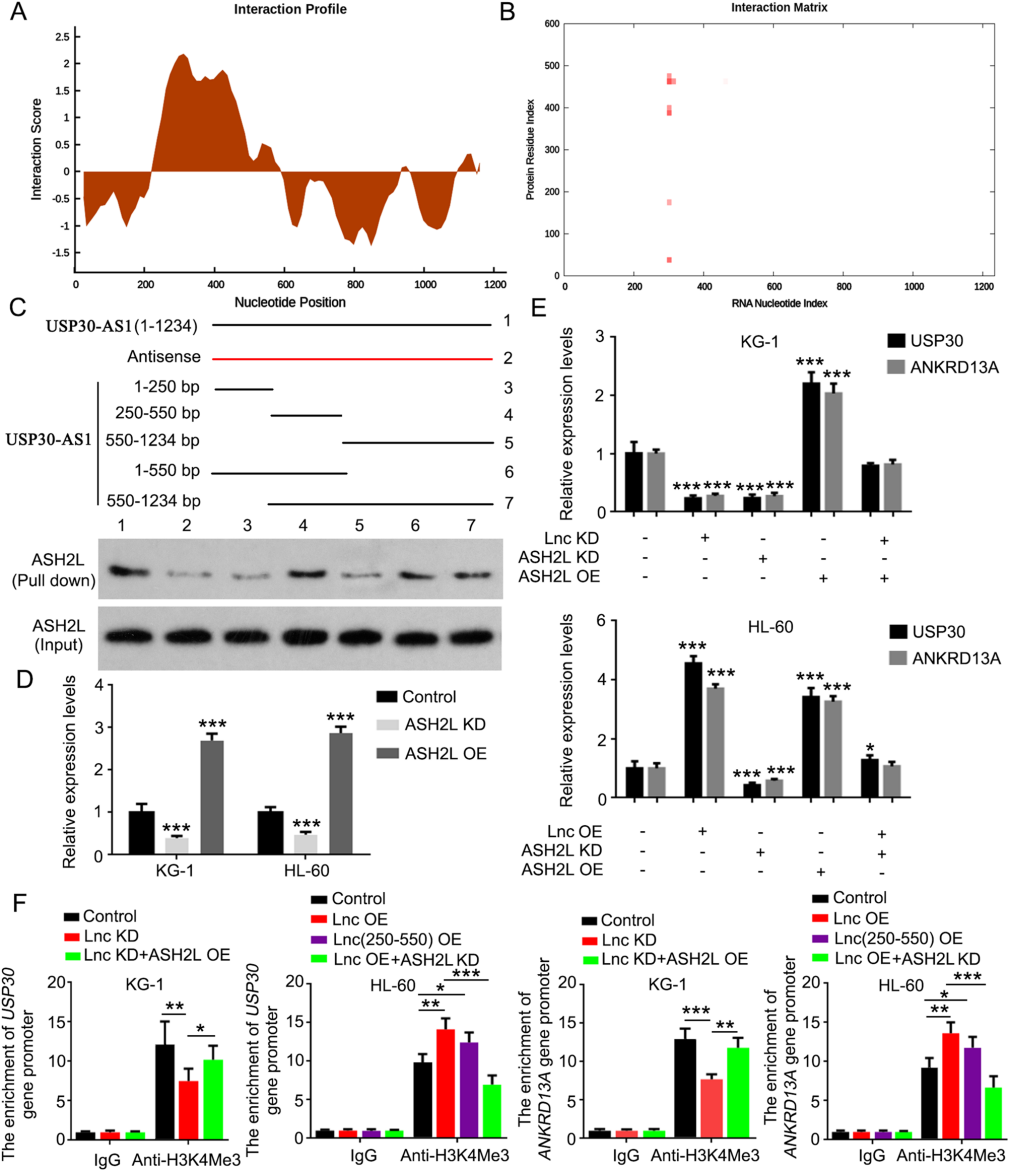
ASH2L is implicated in the *cis* regulation of USP30-AS1 in USP30 and ANKRD13A expression. The interaction between USP30-AS1 and ASH2L is predicted using catRAPID (http://service.tartaglialab.com/update_submission/392876/94a348ba99). **A** Interaction profile; **B** interaction matrix. **C** RNA pull down assay: the full length of USP30-AS1, the antisense of USP30-AS1, as well as the specific regions of the lncRNA were synthesized to pull down ASH2L in HL-60 cells. **D** ASH2L was knocked down or overexpressed in KG-1 and HL-60 cells, followed by PCR assay. **E** Expression of USP30 and ANKRD13A was tested by PCR assay in KG-1 and HL-60 cells with USP30-AS1 KD, USP30-AS1 OE, ASH2L KD or ASH2L OE. **F** We conducted ChIP assay using H3K4me3 as the IP protein in KG-1 and HL-60 cells with USP30-AS1 KD, USP30-AS1 OE, USP30-AS1_250–550_ OE, ASH2L KD or ASH2L OE. Significant differences are indicated by *p < 0.05, **p < 0.01 and ***p < 0.001, *n* = 3

## Discussion

According to results from bioinformatics analysis, lncRNA USP30-AS1 is highly expressed in AML, and both the high expression of USP30-AS1 and low methylation level at Cg03124318 locus of *USP30-AS1* gene promoter are associated with poor prognosis of AML. The methylation level at Cg03124318 was negatively correlated with the expression of USP30-AS1. Therefore, we speculated that low methylation level at Cg03124318 locus leads to the high expression of USP30-AS1 in AML. Further clinical study is needed to determine whether USP30-AS1 expression and the methylation levels at Cg03124318 locus are desirable biomarkers to assess the progression of AML.

USP30-AS1 is an lncRNA transcribed from the antisense chain of the USP30 gene. At present, USP30-AS1 has been studied in bladder urothelial cancer and cervical cancer, which is related to autophagy, proliferation, and apoptosis [18, 29]. Our results showed that when the expression of USP30-AS1 was down-regulated, the viability of KG-1 cells decreased significantly and the apoptotic rate increased. However, forced expression of USP30-AS1 can enhance the viability of HL-60 cells and reduce apoptosis. These results indicated that USP30-AS1 promotes the survival of AML cells. This outcome is in line with survival analysis results, in which high expression of USP30-AS1 was associated with poor prognosis of AML. Therefore, this study first confirmed the cancer-promoting effect of USP30-AS1 in AML, which may be an important lncRNA target in AML treatment.

LncRNA is an important regulatory factor in the human genome, and its molecular regulation mechanism is very complicated [16, 30]. LncRNA can affect DNA methylation, histone modification, and chromosome remodeling through the regulation of transcription and translation levels, and then dynamically control the expression changes of disease-related genes and some important biological processes [16, 30]. A large amount of evidence has shown that DNA methylation dysfunction exists in AML [6]. For example, lncRNA MEG3, as a tumor suppressor of AML, its hypermethylation can lead to a decrease in DNMT3A expression, which in turn inhibits the occurrence of leukemia [31]. Studies have shown that under different stimulation conditions and signaling pathways, some lncRNAs will specifically transcribe, and can directly bind to the DNA of neighboring locations in the chromosome to regulate gene expression, namely, *cis*-regulation [32]. Through high-throughput sequencing studies, we found that USP30-AS1 regulates the expression of multiple genes in AML (including 324 up-regulated and 821 down-regulated genes). These genes are mainly related to the immune regulatory system, epigenetic, transcription, translation, and other regulatory functions. Among them, USP30 and ANKRD13A are genes close to USP30-AS1 gene in chromosome. It is easy to think that the expressions of USP30 and ANKRD13A may be regulated by USP30-AS1 through the *cis*-action. Loss and gain experiments showed that the mRNA and protein expression levels of USP30 and ANKRD13A were positively correlated with the expression of USP30-AS1 in AML. After USP30-AS1 knockdown in AML cells, forced USP30 gene can rescue the decreased cell viability and increased cell apoptosis. However, inhibiting the expression of USP30 in the overexpressed USP30-AS1 cells can inhibit the enhancement of cell viability and reduction of cell apoptosis. USP30 plays an important regulatory role in the occurrence and development of cancer [20, 21]. It can regulate mitochondrial function and lipid metabolism, and affect cell proliferation and apoptosis [20, 21]. Therefore, USP30 mediated the cancer-promoting effects of USP30-AS1 in AML.

In this experiment, we did not found that ANKRD13A has a direct role in the viability and apoptosis of AML cells. However, survival analysis showed that overall survival rate significantly reduced in AML patients with high expression of ANAKRD13A. Therefore, we speculated that ANKRD13A regulates AML progression independent of influencing cell proliferation and apoptosis directly. ANKRD13A is a member of the Ankrin repeat domain protein family, which promotes ubiquitin-dependent internalization of ligand-activated EGF receptor (EGFR) [23]. Many studies have shown that tumor cells can directly or indirectly down-regulate the expression of key molecules that interact with the host immune system [33, 34]. Reduced human MHC HLA-I molecules are a common phenomenon in oncology [26, 35]. Tumor cells with the loss of HLA-I antigens in the cell surface cannot be recognized and attacked by CTL cells, which assists the immune escape of the tumor [26, 35]. In the present research, ANKRD13A also induced HLA-I internalization probably by recognizing lys-63-linked polyubiquitination. Therefore, the up-regulation of ANKRD13A by USP30-AS1 contributed to HLA-I internalization. The regulatory effect of USP30-AS1 on immune escape of AML cells by ANKRD13A/HLA-I is still needed to identify in animal study in the near future.

The regulatory mechanism of lncRNA is very complicated, and its subcellular location largely determines the functional fate of lncRNA [36, 37]. For example, lncRNA in the nucleus mainly regulates chromatin, transcription regulation, and alternative splicing regulation [38, 39]. While in the cytoplasm, lncRNA regulates mRNA stability and translation through multiple mechanisms, such as ceRNA mechanism [40]. In the present study, we found that USP30-AS1 mainly distributed in the nucleus of AML cells, implying that the mechanism of USP30-AS1 in AML was mainly related to chromatin and transcriptional regulation. ChIP analysis showed that the promoters of USP30 and ANKRD13A enriched in all H3K4me1, H3K4me3, and H3K27Ac proteins. However, the lack of USP30-AS1 expression directly affects the enrichment of USP30 and ANKRD13A promoters in H3K4me3 protein. Studies have found that some lncRNAs can regulate gene expression through histone modification or recruitment of transcription factors [16, 41]. H3K4me3 and H3K27Ac modification in the promoter region can change chromatin activity, affect transcriptional regulation, and then control gene expression [41, 42]. Our data indicated that USP30 and ANKRD13A are regulated by USP30-AS1 through affecting histone methylation and acetylation.

Histone methylation is regulated by two counteracting groups of chromatin proteins, namely, PcG and TrxG protein complexes. PcG protein complex suppresses gene transcription activity primarily by inducing tri-methylating histone H3 at lysine 27 (H3K27me3) via the EZH2 [43]. TrxG protein complex maintains the active state of genes through tri-methylation of H3K4 (H3K4me3) [44]. In TrxG protein complex, ASH2L, in combination with RBBP5 and WDR5, stimulates the histone methyltransferase activities of KMT2A, KMT2B, SETD1A, and SETD1B. This study found that USP30-AS1 had strong affinity to ASH2L that belongs to TrxG protein complex. This result is line with the effect of USP30-AS1 on inducing H3K4me3 in the promoter of *USP30* and *ANKRD13A* genes. After knockdown of ASH2L, the *cis*-regulation of USP30-AS1 in USP30 and ANKRD13A expression was impaired. Therefore, the *cis*-regulation of USP30-AS1 is partially associated with ASH2L. It has been reported that some proteins in TrxG complex are recruited by lncRNA to gene promoter to stimulate gene expression. Sun et al. reported that LncRNA GClnc1 acts as a modular scaffold of WDR5 to induce histone modification in the promoter of *GClnc1* gene, an oncogenic in gastric cancer [45]. Butler et al. discovered that ASH2L expression is significantly increased in a subset of AML patients carrying fms-related tyrosine kinase 3 (FLT3) mutations, and the ASH2L expression level is negatively associated with the overall survival rate [46]. The interaction between USP30-AS1 and ASH2L might lead a worse outcome in AML patients.

## Conclusion

In summary, this study confirmed the cancer-promoting effect of USP30-AS1 in AML for the first time. USP30-AS1 can *cis*-regulate the USP30 gene to promote the progression of AML disease. At the same time, USP30-AS1 can also target ANKRD13A to affect the translocation of HLA-Iprotein from cell membrane to cytoplasm, probably leading to the tumor immune escape. USP30-AS1 is mainly involved in the biological process of AML cells through chromatin regulation, and plays a role in promoting cancer. Therefore, USP30-AS1 can be an important lncRNA target for AML treatment.

## Data Availability

The data sets generated/analyzed in the present study are available upon reasonable request from the corresponding author.

## References

[1] Kantarjian H, Kadia T, DiNardo C, Daver N, Borthakur G, Jabbour E, Garcia-Manero G, Konopleva M, Ravandi F (2021). Acute myeloid leukemia: current progress and future directions. Blood Cancer J.

[2] De Kouchkovsky I, Abdul-Hay M (2016). Acute myeloid leukemia: a comprehensive review and 2016 update. Blood Cancer J.

[3] Park JW, Han J (2019). Targeting epigenetics for cancer therapy. ARCH PHARM RES.

[4] Nebbioso A, Tambaro FP, Dell'Aversana C, Altucci L (2018). Cancer epigenetics: moving forward. Plos Genet.

[5] Moore LD, Le T, Fan G (2013). DNA methylation and its basic function. Neuropsychopharmacology.

[6] Yang X, Wong MPM, Ng RK (2019). Aberrant DNA methylation in acute myeloid leukemia and its clinical implications. Int J Mol Sci.

[7] Toyota M, Kopecky KJ, Toyota M, Jair K, Willman CL, Issa JJ (2001). Methylation profiling in acute myeloid leukemia. Blood.

[8] Hu L, Gao Y, Shi Z, Liu Y, Zhao J, Xiao Z, Lou J, Xu Q, Tong X (2019). Dna methylation-based prognostic biomarkers of acute myeloid leukemia patients. Ann Transl Med.

[9] Lin T, Hou H, Chou W, Ou D, Yu S, Tien H, Lin L (2011). Cebpa methylation as a prognostic biomarker in patients with de novo acute myeloid leukemia. Leukemia.

[10] Schmitz SU, Grote P, Herrmann BG (2016). Mechanisms of long noncoding RNA function in development and disease. Cell Mol Life Sci CMLS.

[11] Elcheva IA, Spiegelman VS (2020). The role of *cis*- and *trans*-acting rna regulatory elements in leukemia. Cancers.

[12] Fernando TR, Contreras JR, Zampini M, Rodriguez-Malave NI, Alberti MO, Anguiano J, Tran TM, Palanichamy JK, Gajeton J, Ung NM, Aros CJ, Waters EV, Casero D, Basso G, Pigazzi M, Rao DS (2017). The lncRNA casc15 regulates sox4 expression in RUNX1-rearranged acute leukemia. Mol Cancer.

[13] Cao J (2014). The functional role of long non-coding RNAS and epigenetics. Biol Proced Online.

[14] Statello L, Guo C, Chen L, Huarte M (2021). Gene regulation by long non-coding rnas and its biological functions. Nat Rev Mol Cell Biol.

[15] Yang J, Gould SJ (2013). The *cis*-acting signals that target proteins to exosomes and microvesicles. Biochem Soc Trans.

[16] Long Y, Wang X, Youmans DT, Cech TR (2017). How do lncRNAS regulate transcription?. Sci Adv.

[17] Wang W, Chen T, Zeng Z, Pan Q, Huang W, Han C, Fang K, Sun L, Yang Q, Wang D, Luo X, Sun Y, Chen Y (2020). The lncRNA LAMP5-as1 drives leukemia cell stemness by directly modulating DOT1L methyltransferase activity in MLL leukemia. J Hematol Oncol.

[18] Sun Z, Jing C, Xiao C, Li T (2020). An autophagy-related long non-coding RNA prognostic signature accurately predicts survival outcomes in bladder urothelial carcinoma patients. Aging.

[19] Young M, Hsu K, Lin TE, Chang W, Hung J (2019). The role of ubiquitin-specific peptidases in cancer progression. J Biomed Sci.

[20] Liang J, Martinez A, Lane JD, Mayor U, Clague MJ, Urbé S (2015). Usp30 deubiquitylates mitochondrial parkin substrates and restricts apoptotic cell death. EMBO Rep.

[21] Gu L, Zhu Y, Lin X, Li Y, Cui K, Prochownik EV, Li Y (2018). Amplification of glyceronephosphate *o*-acyltransferase and recruitment of usp30 stabilize DRP1 to promote hepatocarcinogenesis. Cancer Res.

[22] Tanno H, Yamaguchi T, Goto E, Ishido S, Komada M (2012). The Ankrd 13 family of UIM-bearing proteins regulates EGF receptor endocytosis from the plasma membrane. Mol Biol Cell.

[23] Burana D, Yoshihara H, Tanno H, Yamamoto A, Saeki Y, Tanaka K, Komada M (2016). The ankrd13 family of ubiquitin-interacting motif-bearing proteins regulates valosin-containing protein/p97 protein-mediated lysosomal trafficking of caveolin 1. J Biol Chem.

[24] Vanichapol T, Chutipongtanate S, Anurathapan U, Hongeng S (2018). Immune escape mechanisms and future prospects for immunotherapy in neuroblastoma. Biomed Res Int.

[25] Rovatti PE, Gambacorta V, Lorentino F, Ciceri F, Vago L (2020). Mechanisms of leukemia immune evasion and their role in relapse after haploidentical hematopoietic cell transplantation. Front Immunol.

[26] Dhatchinamoorthy K, Colbert JD, Rock KL (2021). Cancer immune evasion through loss of mhc class i antigen presentation. Front Immunol.

[27] Bernard DJ, Courjal F, Maurizis JC, Bignon YJ, Chollet P, Plagne R (1992). Effect of epidermal growth factor in HLA class I and class II transcription and protein expression in human breast adenocarcinoma cell lines. Brit J Cancer.

[28] Tang Z, Li C, Kang B, Gao G, Li C, Zhang Z (2017). Gepia: a web server for cancer and normal gene expression profiling and interactive analyses. Nucleic Acids Res.

[29] Chen M, Chi Y, Chen H, Zhao L (2021). Long non-coding RNA usp30-as1 aggravates the malignant progression of cervical cancer by sequestering microrna-299-3p and thereby overexpressing ptp4a1. Oncol Lett.

[30] Kondo Y, Shinjo K, Katsushima K (2017). Long non-coding RNAS as an epigenetic regulator in human cancers. Cancer Sci.

[31] Li Z, Yang L, Liu X, Wang X, Pan Y, Luo J (2018). The long noncoding RNA MEG3 and its target miR-147 regulate JAK/STAT pathway in advanced chronic myeloid leukemia. EBioMedicine.

[32] Engreitz JM, Haines JE, Perez EM, Munson G, Chen J, Kane M, McDonel PE, Guttman M, Lander ES (2016). Local regulation of gene expression by lncRNA promoters, transcription and splicing. Nature.

[33] Bannister S, Messina NL, Novakovic B, Curtis N (2020). The emerging role of epigenetics in the immune response to vaccination and infection: a systematic review. Epigenetics-US.

[34] Surace AEA, Hedrich CM (2019). The role of epigenetics in autoimmune/inflammatory disease. Front Immunol.

[35] Tanaka K, Tsuchikawa T, Miyamoto M, Maki T, Ichinokawa M, Kubota KC, Shichinohe T, Hirano S, Ferrone S, Dosaka-Akita H, Matsuno Y, Kondo S (2012). Down-regulation of human leukocyte antigen class I heavy chain in tumors is associated with a poor prognosis in advanced esophageal cancer patients. Int J Oncol.

[36] Kopp F, Mendell JT (2018). Functional classification and experimental dissection of long noncoding RNAS. Cell.

[37] Peng W, Koirala P, Mo Y (2017). LncRNA-mediated regulation of cell signaling in cancer. Oncogene.

[38] Cabili MN, Dunagin MC, McClanahan PD, Biaesch A, Padovan-Merhar O, Regev A, Rinn JL, Raj A (2015). Localization and abundance analysis of human lncRNAS at single-cell and single-molecule resolution. Genome Biol.

[39] Sun Q, Hao Q, Prasanth KV (2018). Nuclear long noncoding RNAS: key regulators of gene expression. Trends Genet TIG.

[40] Noh JH, Kim KM, McClusky WG, Abdelmohsen K, Gorospe M (2018). Cytoplasmic functions of long noncoding RNAS. Wiley Interdiscip Rev RNA.

[41] Sarkar D, Leung EY, Baguley BC, Finlay GJ, Askarian-Amiri ME (2015). Epigenetic regulation in human melanoma: past and future. Epigenetics-US.

[42] Wan G, Zhou W, Hu Y, Ma R, Jin S, Liu G, Jiang Q (2016). Transcriptional regulation of lncRNA genes by histone modification in Alzheimer's disease. Biomed Res Int.

[43] Papp B, Müller J (2006). Histone trimethylation and the maintenance of transcriptional ON and OFF states by trxG and PcG proteins. Genes Dev.

[44] Schuettengruber B, Martinez AM, Iovino N, Cavalli G (2011). Trithorax group proteins: switching genes on and keeping them active. Nat Rev Mol Cell Biol.

[45] Sun TT, He J, Liang Q, Ren LL, Yan TT, Yu TC, Tang JY, Bao YJ, Hu Y, Lin Y, Sun D, Chen YX, Hong J, Chen H, Zou W, Fang JY (2016). LncRNA GClnc1 promotes gastric carcinogenesis and may act as a modular scaffold of WDR5 and KAT2A complexes to specify the histone modification pattern. Cancer Discov.

[46] Butler JS, Qiu YH, Zhang N, Yoo SY, Coombes KR, Dent SY, Kornblau SM (2017). Low expression of ASH2L protein correlates with a favorable outcome in acute myeloid leukemia. Leuk Lymphoma.

